# Effects of awareness that food intake is being measured by a universal eating monitor on the consumption of a pasta lunch and a cookie snack in healthy female volunteers^☆^

**DOI:** 10.1016/j.appet.2015.05.034

**Published:** 2015-09-01

**Authors:** J.M. Thomas, C.T. Dourish, S. Higgs

**Affiliations:** a School of Psychology, University of Birmingham, Edgbaston, Birmingham B15 2TT, UK; b P1vital, Manor House, Howbery Park, Wallingford, Oxfordshire OX10 8BA, UK

**Keywords:** Universal eating monitor, UEM, Appetite, Experimenter effects, Eating behaviour, Awareness

## Abstract

To date, there have been no studies that have explicitly examined the effect of awareness on the consumption of food from a Universal Eating Monitor (UEM – hidden balance interfaced to a computer which covertly records eating behaviour). We tested whether awareness of a UEM affected consumption of a pasta lunch and a cookie snack. 39 female participants were randomly assigned to either an aware or unaware condition. After being informed of the presence of the UEM (aware) or not being told about its presence (unaware), participants consumed ad-libitum a pasta lunch from the UEM followed by a cookie snack. Awareness of the UEM did not significantly affect the amount of pasta or cookies eaten. However, awareness significantly reduced the rate of cookie consumption. These results suggest that awareness of being monitored by the UEM has no effect on the consumption of a pasta meal, but does influence the consumption of a cookie snack in the absence of hunger. Hence, energy dense snack foods consumed after a meal may be more susceptible to awareness of monitoring than staple food items.

## Highlights

• Awareness of the presence of a universal eating monitor was manipulated.
• Awareness of food monitoring did not affect amount of food consumed.
• Awareness of food monitoring reduced the rate of cookie consumption.

## Introduction

Measuring food intake within laboratory settings can be a challenge and it has been suggested that consumption in a laboratory may not be representative of normal eating behaviour (Meiselman, 1992). One concern about laboratory based assessment of eating is that if participants are aware that their intake is being monitored, this might affect how much food is consumed.

There is evidence that when participants are directly observed by a researcher who is present in the same room, they consume less food than when an experimenter is not present (Roth, Herman, Polivy, & Pliner, 2001). This inhibitory effect of observation on eating also extends to situations when the experimenter is not in the same room, but participants believe the experimenter will know how much food they have consumed (Polivy, Herman, Hackett, & Kuleshnyk, 1986). More recently, Robinson, Kersbergen, Brunstrom, and Field (2014) conducted two experiments to examine how awareness of food intake monitoring affects eating behaviour. In the first study, they found that when participants believed their food intake would be monitored, the majority of participants indicated that they would eat less food as a consequence. In the second study, when participants were explicitly informed that their food intake would be monitored, they consumed less in a taste test than when they were not given information about monitoring of intake. Given the evidence that participants may change their eating behaviour in response to knowing that their intake is being monitored, it is important to extend our understanding of how awareness of consumption monitoring affects eating in the laboratory.

Appetite researchers usually use cover stories and paradigms designed to reduce awareness that food intake is being monitored to mitigate potential effects on intake. For instance, the disguised taste-test paradigm requires participants to provide sensory ratings of foods. However, the sensory ratings are a cover story, and the true aim is to examine the amount of food consumed (Higgs, 2002). Nevertheless there is evidence that participants in laboratory studies may believe that their intake is being measured, even when they are told that it is not being monitored (Robinson et al., 2014).

Most research on awareness of monitoring has been conducted on intake of highly palatable energy dense snack foods (e.g. cookies: Polivy et al., 1986; Robinson et al., 2014; Roth et al., 2001). Comparatively less work has investigated whether awareness affects the intake of staple foods, lunches and subsequent snacks. Westerterp-Plantenga et al. (1990) and Westerterp-Plantenga, Wouters, and ten Hoor (1991) reported that participants who were made aware that their consumption of a pasta lunch would be monitored did not eat differently from participants who were unaware of the monitoring procedure. However, a fixed portion of pasta was provided to participants, which might have limited the ability to detect differences between groups. Nevertheless, the results of Westerterp-Plantenga et al. (1990) are particularly interesting because they used a universal eating monitor (UEM), a device developed to measure food intake in a covert manner (Kissileff, Klingsberg, & Van Itallie, 1980). The UEM comprises a concealed balance which is interfaced to a computer that records weight every few seconds. By serving food to participants on a plate placed on a mat covering the balance, it is possible to record within-meal eating behaviour. It is important to ascertain whether awareness of a UEM affects eating behaviour because inadvertent movement of the balance by participants can lead to loss of data. From our own work and that of others (Hubel, Laessle, Lehrke, & Jass, 2006; Thomas, Dourish, Tomlinson, Hassan-Smith, & Higgs, 2014), it has been shown that testing unaware participants who may for example, accidentally lean on the scales, can lead to losses of up to 26% of study data. If awareness of the UEM does not affect intake, then making participants aware of its presence could potentially prevent such data loss while avoiding problems with demand effects.

In this study we tested whether explicit awareness of the UEM would affect intake of a pasta lunch (staple food item), and a subsequent cookie snack (palatable, energy dense food item). The use of the UEM allows us to examine whether awareness of monitoring affects the total amount of food consumed, the microstructure of a meal and within meal appetite ratings. Participants had ad-libitum access to a pasta meal, followed twenty minutes later by ad-libitum access to a chocolate chip cookie snack. Participants in the aware condition were made explicitly aware of the presence of the UEM, while those in the unaware condition were not. It was hypothesised that awareness of the UEM would decrease the amount of cookies consumed, but have no effect on the amount of pasta that was eaten.

## Materials and methods

### Participants

A total of 72 female student volunteers were recruited from the School of Psychology at the University of Birmingham. During testing, 3 participants in the unaware condition became aware of the UEM, while 30 participants (25 unaware and 5 aware) accidentally leaned on the UEM balance during their test session, triggering an error with the software which prevented accurate measurement of subsequent eating behaviour. Therefore, 39 participants successfully completed testing and their data were used for analysis. The 39 participants had a mean age of 19.7 years (SEM 0.2) and a mean body mass index (BMI) of 21.8 (SEM 0.4). Reimbursement for participation in the study took the form of course credits or a £10 payment. Informed consent was obtained from participants and ethical approval was provided by the University of Birmingham Research Ethics Committee. The study was conducted in accordance with Good Clinical Practice and the ethical standards laid down in the 1964 Declaration of Helsinki. Participants were not recruited if they: had food allergies; smoked cigarettes; took medication that affected appetite; were diabetic or had participated in a previous study using a UEM. All of these were assessed via questionnaire in the laboratory.

### Design

A between-subjects design was used with a single factor of awareness with two levels: aware and unaware. Participants were randomly allocated to one of these conditions and order of testing within sessions was counterbalanced so that half of the participants completed a batch of questionnaires followed by a computer task, while the other half had the order reversed. Based on an awareness study by Roth et al. (2001) and a UEM study by Yeomans (1996), effect sizes were calculated (Cohen's d = 0.97 and 1.00, respectively), and power analyses were conducted, showing that at least 18 participants were required per group to detect an effect (80% power; *p* < 0.05).

### Universal eating monitor (UEM)

Test meals were served on a Sussex Ingestion Pattern Monitor (SIPM), a validated UEM (Yeomans, 2000). This consisted of a balance (Sartorius Model CP4201, Sartorius Ltd., Epsom, UK; 0.1 g accuracy) placed underneath, but protruding through, the surface of a table. A placemat on the table was used to hide the balance from the participants' view. The balance was connected to a laptop computer and relayed balance weights every 2 seconds.

#### Pasta lunch

Based on our previous work (Thomas et al., 2014), dishes filled with 220 g (253 kcal) of pasta were set on the placemat. Each time the participant ate 50 g of pasta, the SIPM software (version 2.0.13) interrupted the participant with instructions to complete computerised VAS ratings (hunger, fullness and pleasantness of the pasta). After consuming 150 g, participants were interrupted and provided with a fresh dish of 220 g of pasta. Participants were asked to eat until they felt ‘comfortably full’. The lunch consisted of pasta shells in a tomato and herb sauce (Sainsbury's UK), served at 55–60 °C.

#### Cookie snack

Bowls containing 80 g (390 kcal) of cookie pieces were set on the placemat. Each time the participant ate 10 g of cookie pieces, the SIPM software interrupted the participant with instructions to complete VAS ratings as described above for pasta. After consuming 60 g, participants were interrupted and provided with a fresh bowl containing 80 g of cookie pieces. Participants were asked to eat until they felt ‘comfortably full’. The cookies were Maryland Chocolate Chip Cookies, with each cookie being broken into 6–7 pieces. This approach was designed to reduce the likelihood that participants could track the number of cookies they ate (Higgs & Woodward, 2009).

### Stop signal reaction time task (SSRT)

Behavioural impulsivity has been reported to affect the consumption of food (Guerrieri et al., 2007), hence, the SSRT was included to ensure that there were no differences between groups on this measure. The SSRT (as described in Verbruggen, Logan, & Stevens, 2008) involves presenting participants with either a square or a circle shape on a screen that they are required to identify. On no-signal trials, a shape is presented and participants respond by identifying the shape. On stop-signal trials, an auditory stop signal alerts participants to withhold making a response to the presentation of the shape. The task consists of 32 practice trials followed by 192 experimental trials and takes 20 minutes. Calculation of the stop signal reaction time provides a measure of inhibition of response (behavioural impulsivity).

### Procedure

Participants arrived in a pre-meal state having refrained from eating for 2 hours prior to arrival. They completed a consent form, and were screened using a lifestyle questionnaire which collected demographic information. After this, they completed the stop signal reaction time task as a measure of response inhibition and a series of questionnaires. The questionnaires comprised the Barratt Impulsivity Scale (BIS – Patton, Stanford, & Barratt, 1995) and the Behavioural Inhibition/Approach Scales (BIS/BAS – Carver & White, 1994) as additional measures of impulsivity. Participants also completed the Three Factor Eating Questionnaire (TFEQ – Stunkard & Messick, 1985), as a measure of dietary restraint and tendency towards disinhibition, and the Power of Food Scale (PFS, Lowe et al., 2009), as a measure of sensitivity to food to ensure no differences between groups. A breakfast questionnaire was used to ensure that no food was eaten within the previous two hours, and participants completed a set of baseline visual analogue scales (VAS) for rated mood and appetite items on a scale from 0 to 100 mm (0 mm anchor = not at all, 100 mm anchor = extremely): ‘alertness’; ‘disgust’; ‘drowsiness’; ‘light-headed’; ‘anxiety’; ‘happiness’; ‘nausea; ‘sadness’; ‘withdrawn’; ‘faint’; ‘hungry’; ‘full’; ‘desire to eat’ and ‘thirst’.

Participants were taken to a room containing the UEM. Those in the aware condition were shown that there was a balance underneath the table. They were told that the balance would record the weight of their bowl and food as they ate during the meal and that this information would be stored on the computer it was connected to for later analysis. Those in the unaware condition were not given this information. After they had been given instructions regarding the procedure (i.e. that they could eat as many bowls of pasta as they wished until they were comfortably full), the participants were asked to eat a pasta lunch, as described above. After they had finished their lunch, participants immediately completed another set of VAS, and were then given a 20 minute rest period in another room, where they were offered a home furniture magazine to read. Participants then completed another set of VAS immediately before being taken back to the UEM where they were offered a snack of cookies and asked to eat as much as they wished. Following this snack, participants completed a final set of VAS, and had their height and weight taken for BMI calculation. To assess awareness of the UEM all participants were then asked what they thought the study was about, and whether they had noticed the balance at any point, or whether they thought their intake was being recorded during the study. After this, participants were debriefed, thanked for their time, and compensated with course credits or a cash payment.

### Data analysis

#### General

Effects of awareness were determined with independent t-tests. Repeated-measures analysis of variance (ANOVA) was used to examine temporal effects and interactions with awareness. Only significant effects of awareness, or temporal interactions with awareness, were followed up with planned comparisons and all post-hoc t-tests used the Bonferroni correction. Violations of sphericity were addressed using the Greenhouse–Geisser correction.

#### VAS

Principal components analysis (PCA) was run with varimax rotation yielding 3 factors (items loaded >0.5) with eigenvalues >1, accounting for 59.99% of the variance. Factors included: appetite (hunger, fullness and desire to eat); negative effects (sadness, nausea, disgust, faint, withdrawn, lightheaded) and arousal (alertness, happiness, drowsiness). Anxiety and thirst did not load onto these factors and were analysed separately.

#### UEM

The following measures were calculated for UEM data: amount eaten, time spent eating, eating rate and pause between mouthfuls. The first three measures are standard measures of eating behaviour in microstructural studies, while the latter is a novel measure. It was included because it provides data on the time taken between mouthfuls and also provides useful data on frequency of mouthfuls. For instance, if time is constant, then shorter pauses equate to more mouthfuls and vice versa.

## Results

### Manipulation check

At the end of the study all participants were asked: “Were you aware during testing, that there were scales underneath the table, and that the weight of the food you were eating was recorded by the computer?” Of the 39 participants who were included, 20 had remained aware and 19 had remained unaware during testing. These participants were also asked subsequently: “Did you think the amount of pasta or cookies you ate was being monitored in any other way?” None of the participants thought the investigator might be weighing their food or monitoring their intake in any other way.

### Baseline measures and visual analogue scales

To ensure there were no group differences in demographics and behaviours which might affect food consumption (e.g. impulsivity, food sensitivity, cognitive restraint, etc.) all scores were analysed using independent t-tests comparing aware and unaware conditions. There were no significant differences for all scales and subscales: BMI; Age; TFEQ; BIS 11; PFS; BIS; BAS; and SSRT (all *p* > 0.05; Table 1).

VAS data were analysed by condition (aware vs. unaware) and by time (pre-pasta, post-pasta, pre-cookies and post-cookies). For appetite, arousal, anxiety and thirst there were main effects of time (all *p* < 0.01; means displayed in Table 2) which were not analysed further, but there were no effects of condition (see Table 2 for means) and no interactions (all *p* > 0.05). For negative effects, there was a main effect of time (*F* (3, 99) = 18.48; *p* < 0.001), no effect of condition (*F* (1, 33) = 0.10; *p* > 0.05) and a significant interaction between condition and time (*F* (3, 99) = 3.36; *p* < 0.05). T-tests comparing the effect of unaware versus aware did not reveal any significant differences at any time points; pre pasta (18 mm vs. 12 mm; *p* > 0.05); post pasta (9 mm vs. 10 mm; *p* > 0.05); pre cookies (9 mm vs. 8 mm; *p* > 0.05); post pasta (8 mm vs. 8 mm; *p* > 0.05).

### Universal eating monitor

Independent t-tests were used to analyse the following measures of pasta and cookie consumption by condition (unaware vs. aware): total amount eaten; time spent eating; pause between mouthfuls and eating rate. For the pasta lunch, there was no significant effect of awareness on any of the UEM measures (all *p* < 0.05; see Table 3). For the cookie snack, there was a main effect of awareness for eating rate as participants in the aware condition ate cookies at a slower rate than those in the unaware condition (10.2 vs. 13.4 g/min; *t* (37) = 2.39, *p* < 0.05; Table 3). There was no effect of awareness on any other measures of cookie consumption (all p > 0.05 – Table 3). However, it is interesting to note that there is a trend towards an increase in pause between mouthfuls of cookies for the aware condition (compared to unaware), which is likely to be responsible for the significant decrease in cookie eating rate in the aware condition.

### Computerised within-meal VAS

**Fig. 1:**
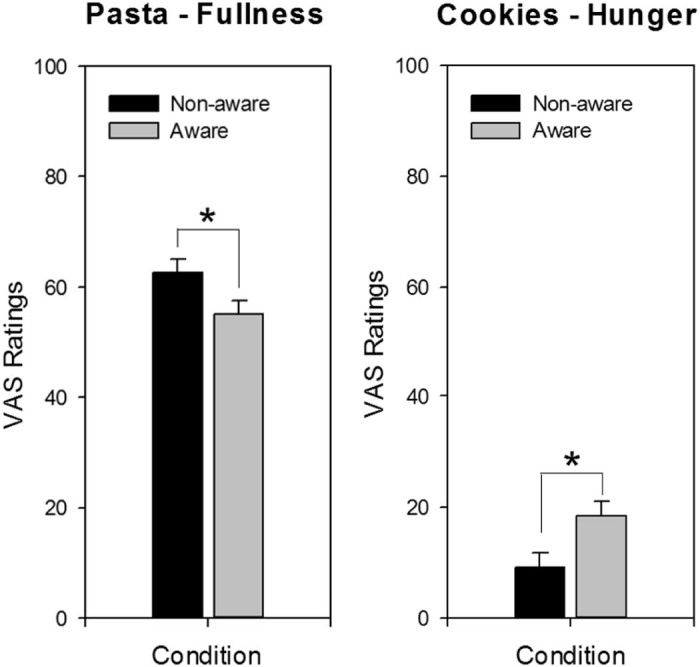
Mean fullness and hunger ratings while consuming pasta and cookies, respectively. Rated fullness while eating pasta (left) was significantly decreased when participants were aware (vs. unaware); mean rated hunger while eating cookies (right) was significantly increased when participants were aware. *p < 0.05.

VAS ratings made during the meal, were used to calculate mean hunger, fullness and pleasantness ratings. These were analysed with independent t-tests by condition (unaware vs. aware). For pasta, rated fullness was significantly lower in the aware condition compared to the unaware condition (55 mm vs. 63 mm; *t* (37) = 2.12, *p* < 0.05; see Fig. 1), and there was a trend for greater hunger in the aware versus unaware condition (42 mm vs. 35 mm; *t* (37) = −1.78, *p* = 0.08). Rated pleasantness did not significantly differ between conditions (aware = 70 mm vs. unaware = 68 mm; *t* (37) = −0.50, *p* > 0.05). For cookies, hunger was significantly higher in the aware group versus unaware (18 mm vs. 9 mm; *t* (37) = −2.48, *p* < 0.05; Fig. 1). However, neither rated fullness nor pleasantness of the cookies, differed by condition (both *p* > 0.05).

## Discussion

Awareness of the UEM significantly reduced the eating rate of cookies, but had no effect on the amount of cookies or pasta eaten, nor on any other measures of consumption. Participants in the aware condition reported lower levels of fullness while consuming pasta, and higher levels of hunger when consuming the cookies. Hence, awareness of food monitoring via a UEM had limited effects on eating behaviour. These data suggest that studies using comparable UEM paradigms and participant populations might consider making their participants aware of the presence of a UEM to mitigate potential problems with loss of data due to accidental movement of the balance.

The decrease in cookie eating rate but not pasta eating rate may be due to a number of reasons. It might be that individuals were more concerned about being seen to eat “forbidden” foods such as cookies (vs. pasta) and reduced their eating rated to present a positive impression (Herman, Roth, & Polivy, 2003; Macht, Gerer, & Ellgring, 2003). It is also possible that these effects may be related to the type of eating episode (lunch vs. snack), and this is worth investigating in future work.

Reported hunger was low while consuming the cookies (but not pasta), and has also been reported to be low in other studies in which awareness effects were observed (Robinson et al., 2014). Hence, it might also be that foods consumed in the absence of hunger are more susceptible to the effects of awareness. It is interesting that fullness ratings decreased while consuming pasta and hunger ratings increased while consuming the cookies. This may reflect an attempt to present a positive impression, by appearing to terminate intake before reaching a high level of fullness (pasta), or while still hungry (cookies), although this is speculative.

The manner in which the cookies were provided might also have affected food intake. In the study by Robinson et al. (2014) the participants were presented with whole cookies. However, we broke the cookies into several pieces to make it more difficult for individuals to track the amount of food they consumed. Hence, it is possible that participants in the aware condition wanted to give the impression that they were consuming a small amount of cookies, but had difficulty tracking how much they had eaten. In support of this idea, it has been shown that participants eat more of the same food when it is presented amorphously, as numerous small pieces/parts, than as a whole item, and is likely due to difficulty in tracking intake (Chang et al., 2012).

Interestingly, the total amount of cookies consumed was not affected by awareness of monitoring. Perhaps the use of automated technology has less of an impact on eating behaviour than the knowledge that a researcher will be examining and weighing the food consumed. Alternatively, it may be that as the participants are regularly interrupted by the UEM software during the meal to make VAS ratings, this increases awareness of how much food is being consumed, and thereby reduces the impact of the explicit awareness manipulation. A comparison of the effects of automated versus experimenter monitored intake under the same conditions is required to further explore these issues.

In the present study we recruited only female participants. This was based on a previous study using a UEM in which some male participants engaged in “competitive eating” and consumed very large amounts of pasta (Thomas et al., 2014). Therefore, it remains to be investigated whether men would behave similarly when aware or unaware of intake monitoring. In addition it will be important to examine how individual characteristics such as BMI and dietary restraint interact with awareness, since dieters and obese participants may be more concerned about issues of self-presentation than lean non-dieters (Younger & Pliner, 1976). Finally, the results of this study should be considered in relation to the specific eating situation investigated. While we found no effects of awareness of the UEM on total food intake, the limited effects we identified on meal microstructure measures are consistent with previous observations of a potentially important effect of awareness of monitoring when participants are offered high energy dense snack foods to eat (Robinson et al., 2014). However, these effects require replication in a more representative sample (e.g. males and females, wider range of BMI, etc.).

To date, there have been relatively few investigations of the influence of participant awareness of food intake measurement on eating behaviour, and it is clear that a better understanding of these effects will enable improved design and interpretation of results in future studies. A caveat is that these results were obtained with females eating two test foods from a UEM. Thus, the effects might not translate to other populations or food types, and requires further investigation.

### Conclusions

Awareness of the presence of a UEM reduced the rate of consumption of a cookie snack, but had no effect on consumption of a pasta lunch. In addition, participants who were aware of the UEM reported lower levels of fullness while consuming pasta and higher levels of hunger when consuming the cookies. Hence, awareness of this type of monitoring of food intake had relatively limited effects, particularly on consumption of staple foods.

## Figures and Tables

**Table 1:** Mean baseline scores for unaware and aware groups (standard error of the mean).

Measure	Unaware	Aware	t Value	*p* Value
BMI	21.9 (0.5)	21.8 (0.5)	0.06	0.95
Age	20.0 (0.4)	19.4 (0.2)	1.39	0.17
TFEQ cognitive restraint	8.3 (1.0)	9.1 (1.2)	−0.51	0.62
TFEQ disinhibition	8.8 (0.7)	7.1 (0.8)	1.67	0.10
TFEQ hunger	7.3 (0.7)	7.2 (0.7)	0.12	0.90
BIS 11	69.1 (2.7)	66.6 (2.1)	0.76	0.45
PFS	40.8 (2.5)	43.7 (2.4)	−0.84	0.41
BIS	23.2 (1.0)	24.1 (0.6)	−0.72	0.48
BAS drive	10.4 (0.5)	11.0 (0.5)	−0.81	0.42
BAS funseeking	12.0 (0.4)	11.7 (0.5)	0.32	0.75
BAS reward responsiveness	16.7 (0.4)	17.4 (0.4)	−1.17	0.25
SSRT (milliseconds)	233.0 (7.0)	233.8 (5.0)	−0.10	0.92

*Notes:* BMI, Body Mass Index; TFEQ, Three Factor Eating Questionnaire; BIS 11, Barratt Impulsiveness Scale; PFS, Power of Food Scale; BIS, Behavioural Inhibition Scale; BAS, Behavioural Activation Scale; SSRT, Stop Signal Reaction Time.

**Table 2:** VAS measures separated by time and by condition (standard error of the mean).

VAS measure	Main effect of time				Main effect of condition	
	Pre-pasta	Post-pasta	Pre-cookies	Post-cookies	Unaware	Aware
Appetite	73 (2.6)	15 (2.0)	17 (2.2)	10 (1.7)	28 (2.1)	31 (2.1)
Negative effects	15 (2.3)	10 (1.5)	9 (1.4)	8 (1.1)	11 (2.3)	10 (2.3)
Anxiety	21 (3.5)	11 (2.3)	9 (1.6)	9 (1.7)	13 (3.2)	13 (3.1)
Arousal	54 (2.5)	62 (2.4)	58 (2.1)	60 (2.1)	61 (2.7)	56 (2.6)
Thirst	53 (4.4)	44 (3.8)	37 (4.2)	34 (4.2)	42 (5.5)	43 (5.4)

**Table 3:** UEM measures for pasta and cookies, split by unaware versus aware groups (standard error of the mean).

Measure	Unaware	Aware	t Value	*p* Value
Pasta				
Amount eaten (grams)	361.6 (20.6)	365.1 (30.7)	−0.10	0.92
Time spent eating (seconds)	422.4 (62.4)	386.8 (21.2)	0.55	0.58
Pause between mouthfuls (seconds)	8.3 (0.9)	8.6 (0.4)	−0.29	0.77
Amount eaten per minute (g/min)	63.0 (4.8)	58.9 (3.5)	0.69	0.49
Cookies				
Amount eaten (grams)	40.3 (3.5)	36.7 (4.3)	0.64	0.52
Time spent eating (seconds)	227.1 (25.0)	274.4 (27.1)	−1.28	0.21
Pause between mouthfuls (seconds)	12.4 (2.2)	16.3 (1.6)	2.39	0.16
Amount eaten per minute (g/min)	13.4 (0.8)	10.2 (1.0)	−1.45	0.02*

* *p* < 0.05.
